# Human Spinal Cord GABAergic Neural Progenitor Cell

**DOI:** 10.1111/cpr.70152

**Published:** 2025-12-30

**Authors:** Jia Xu, Xiaoqing Zhang, Aijin Ma, Jie Hao, Tianqing Li, Boqiang Fu, Lixiang Ma, Yan Liu, Peng Xiang, Kun Qian, Xiaohua Han, Yajie Li, Lijun Zhu, Qiyuan Li, Qiang Wei, Tingting Wu, Lei Wang, Jiani Cao, Ka Li, Hongling Zhao, ShuaiShuai Niu, Baoyang Hu, Tongbiao Zhao, Hong Chen

**Affiliations:** ^1^ Tongji Hospital, Tongji Medical College Huazhong University of Science and Technology Wuhan China; ^2^ Tongji University School of Medicine Shanghai China; ^3^ Beijing Technology and Business University Beijing China; ^4^ Standards Committee, Chinese Society for Cell Biology Shanghai China; ^5^ National Stem Cell Resource Bank Beijing China; ^6^ Institute of Zoology Chinese Academy of Sciences Beijing China; ^7^ Institute of Primate Translational Medicine Kunming University of Science and Technology Kunming China; ^8^ China Institute of Metrology Beijing China; ^9^ School of Basic Medicine Fudan University Shanghai China; ^10^ School of Pharmacy Nanjing Medical University Nanjing China; ^11^ Sun Yat‐Sen University Guangzhou China; ^12^ Institute of Science and Technology Information of China Beijing China; ^13^ Shenzhen Medical Academy of Research and Translation Shenzhen China; ^14^ Institute of Laboratory Animal Science, CAMS Beijing China; ^15^ National Institutes for Food and Drug Control Beijing China; ^16^ Zephyrm Biotechnologies Co., Ltd. Beijing China; ^17^ Zhongshan Hospital Fudan University Shanghai China; ^18^ Beijing Institute for Stem Cell and Regenerative Medicine Beijing China

‘Human spinal cord GABAergic neural progenitor cell’ is the latest set of guidelines on human spinal cord GABAergic neural progenitor cells in China, jointly drafted and agreed upon by experts from the Standard Committee of Chinese Society for Cell Biology. This standard specifies requirements for human spinal cord GABAergic neural progenitor cells, including the technical requirements, test methods, test regulations, instructions for use, labelling requirements, packaging requirements, storage and transportation requirements and waste disposal requirements. This standard is applicable for the quality control of human spinal cord GABAergic neural progenitor cells, whether derived from human tissues or differentiated/transdifferentiated from stem cells. It was originally released by the Chinese Society for Cell Biology on 28 October, 2024. We hope that the publication of these guidelines will promote institutional establishment, acceptance and execution of proper protocols, thereby accelerating the international standardisation of human spinal cord GABAergic neural progenitor cells for various applications.

## 1 Scope

This document specifies the general requirements, technical requirements, detection methods, inspection rules, instructions for use, labelling, transportation and storage of human spinal cord GABAergic neural precursor cells.

This document is applicable to the detection of human spinal cord GABAergic neural precursor cells that are isolated from human tissues or obtained through stem cell differentiation or transdifferentiation.

## 2 Normative References

The contents in the following documents constitute indispensable clauses of this document through normative references in the text. Among them, for dated references, only the version corresponding to the date is applicable to this document; for undated references, the latest version (including all amendments) is applicable to this document.

Pharmacopoeia of the People's Republic of China (2020 edition, Volume III)

National clinical laboratory operating procedures

WS 213 diagnosis of hepatitis C

WS 273 diagnosis of syphilis

WS 293 diagnostic criteria for AIDS and HIV infection

## 3 Terms and Definitions

The following terms and definitions apply to this document.

### 3.1 Human Spinal Cord GABAergic Neural Progenitor Cell

Cells with the potentials to differentiate into GABA interneurons and astrocytes, which can be obtained through direct isolation from spinal cord tissues, directional differentiation from human pluripotent stem cells, or transdifferentiation.

### 3.2 Human Spinal Cord GABAergic Interneuron

An inhibitory interneuron located in the human dorsal spinal cord that regulates the balance of excitation and inhibition within neural networks.

## 4 Abbreviations

The following abbreviations apply to this document.

- EBV: Epstein Barr virus;
- HBV: Hepatitis B virus;
- GABA: γ—aminobutyric acid;
- GAD: Glutamic acid decarboxylase;
- HCMV: Human cytomegalovirus;
- HCV: Hepatitis C virus;
- HIV: Human immunodeficiency virus;
- HTLV: Human T‐lymphotropic virus;
- hPSC: Human pluripotent stem cell;
- STR: Short tandem repeat;
- TP: *Treponema pallidum* .

## 5 General Requirements

### 5.1 Raw Materials

The acquisition of raw materials and their use in research shall comply with domestic recognised ethics and local laws and regulations.

According to the purpose of use, cell research and production organisations shall establish corresponding donor evaluation criteria.

### 5.2 Process and Information Management

The key factors affecting product quality in the process of raw material acquisition, preparation, detection, transportation and storage of cells should be recorded.

The minimum retention time of records shall be specified to ensure the integrity of records and the safety of record storage.

## 6 Technical Requirements

### 6.1 Cell Morphology

Under the condition of suspension culture, it has a dense neurosphere‐like morphology, good transparency and smooth and regular edges.

### 6.2 Cell Marker Proteins

The positive rate of human spinal cord marker HOXB4 should be ≥ 80%, the positive rate of human spinal cord GABAergic neural precursor cell marker ptf1a should be ≥ 80%, and the positive rate of ascl1 should be ≥ 80%.

### 6.3 Cell Viability

The survival rate of unfrozen cells shall be ≥ 80%, and the survival rate of frozen cells after resuscitation shall be ≥ 50%.

### 6.4 Cell Function Indicators

When human spinal cord GABAergic neural precursor cells continue to form adult spinal cord GABAergic interneurons in culture, the positive rate of GABA should be ≥ 70%, and the positive rate of gad65/67 (glutamate decarboxylase 65/67, the key enzyme of GABA synthesis) should be ≥ 70%.

According to the whole cell patch clamp electrophysiological detection of human spinal cord GABAergic interneurons, more than 50% of the cells should have the tonic discharge pattern characteristic of spinal cord GABA neurons.

### 6.5 Karyotype

The normal karyotype shall be 46, XX or 46, XY.

### 6.6 Microorganisms

Fungi, bacteria, mycoplasma, HIV, HBV, HCV, HTLV, EBV, HCMV, TP shall be negative.

### 6.7 Bacterial Endotoxin

Endotoxin should be < 5.0 EU/mL.

### 6.8 2STR Identification

It shall conform to the human standard STR map, be consistent with the donor cells, and be free of impurity cell contamination.

## 7 Inspection Method

### 7.1 Cell Morphology

Under the condition of in vitro culture, the cell structure can be observed using light microscope.

### 7.2 Cell Marker Proteins

The method is described in Appendix A.

### 7.3 Cell Viability

The method is described in Appendix B.

### 7.4 Cell Function Indicators

GABA and gad65/67 are examined according to the method in Appendix B. Refer to the method in Appendix C to test the electrophysiological function.

### 7.5 Karyotype

According to the ‘preparation and quality control of animal cell matrix for the production and verification of biological products’ in the Pharmacopoeia of the People's Republic of China (2020 edition, Volume III).

### 7.6 Microorganisms

#### 7.6.1 Bacteria and Fungi

The ‘1101 sterility test method’ in the Pharmacopoeia of the People's Republic of China (2020 Edition, Volume III) shall be followed.

#### 7.6.2 Mycoplasma

The ‘3301 Mycoplasma test method’ in the Pharmacopoeia of the People's Republic of China (2020 edition, Volume III) shall be followed.

#### 7.6.3 HBV

The nucleic acid test method in the National Guide to Clinical Laboratory Procedures shall be followed.

#### 7.6.4 HCV

HCV shall be tested in accordance with the WS 213 nucleic acid method.

#### 7.6.5 HIV

HIV shall be tested in accordance with the WS 293 nucleic acid method.

#### 7.6.6 HTLV

HTLV shall be tested by the nucleic acid method in the National Guide to Clinical Laboratory Procedures.

#### 7.6.7 EBV

The nucleic acid test method in the National Guide to Clinical Laboratory Procedures shall be followed.

#### 7.6.8 HCMV

The nucleic acid test method in the National Guide to Clinical Laboratory Procedures shall be used.

#### 7.6.9 TP

TP shall be tested in accordance with the WS 273 nucleic acid method.

### 7.7 Bacterial Endotoxin

The ‘1143 bacterial endotoxin test method’ in the Pharmacopoeia of the People's Republic of China (2020 Edition, Volume III) shall be followed.

## 8 Inspection Rules

### 8.1 Sampling Method and Quantity

In a production cycle, products prepared from the same batch, source, generation and method are one batch.

Randomly select three minimum packaging units from the same batch of products.

### 8.2 Qualification Rate Inspection

Each batch of products shall be inspected and attached with inspection report.

Inspection items shall include all items specified in Section 6.

### 8.3 Recheck and Inspection

Fitting for purpose, the professional cell testing institution/laboratory should carry out recheck and inspection.

### 8.4 Judgement Rules

All the ex‐factory inspection items comply with the provisions of Section 6 and are judged as qualified products. If one or more items fail to meet the requirements of this document, it will be judged as unqualified.

All the rechecked inspection items meet the provisions of Section 6 and are judged as qualified products; if one or more items fail to meet the requirements of this document, it will be judged as unqualified.

## 9 Instructions for Use

It shall at least include the following contents:

- Name;
- Generation;
- Quantity;
- Production date;
- Production batch number;
- Production organisation;
- Storage conditions;
- Transportation conditions;
- Contact information;
- Method of use;
- Executive standard number;
- Production address;
- Postal code;
- Precautions.

*Note*: Provide endotoxin results according to user requirements.

## 10 Label

It shall at least include the following contents:

- Name;
- Generation;
- Quantity;
- Production batch number;
- Production organisation;
- Date of manufacture.

## 11 Transportation and Storage

### 11.1 Transportation

According to the use requirements of cells, select appropriate transportation methods and conditions to ensure the biological characteristics, safety, stability and effectiveness of cells.

Factors such as cell characteristics, containers carrying cells, transportation routes, transportation conditions, transportation equipment, transportation methods, transportation risks and safeguard measures shall be considered for the transportation of cells.

The control of transportation conditions shall include but not limited to temperature range, oscillation, pollution‐free, equipment performance and appropriate packaging, etc.

Corresponding accompanying inspection documents and technical guidance documents shall be provided according to user requirements

Packages in transit should be checked if necessary, and new freezing sources (such as dry ice or liquid nitrogen) should be added if possible to maintain the appropriate transport temperature.

### 11.2 Storage

Optimised freezing procedures and methods should be adopted to minimise damage to cells during freezing and thawing to ensure that their normal functions are not affected by freezing and thawing.

The cryopreservation information of cells shall be recorded, including but not limited to:

- Name;
- Batch number;
- Quantity;
- Passage times;
- Freezing date;
- Cryopreservation solution components;
- Operator name.

Cell storage conditions shall be recorded, including but not limited to:

- Storage conditions;
- Storage date;
- Storage life;
- Storage personnel.

## Author Contributions

Hong Chen and Jia Xu contributed to conception and design. Baoyang Hu, Xiaoqing Zhang, Tongbiao Zhao, Aijin Ma, Jie Hao, Tianqing Li and Boqiang Fu revised the manuscript. Lixiang Ma, Yan Liu, Peng Xiang, Kun Qian, Xiaohua Han, Yajie Li, Lijun Zhu, Qiyuan Li, Qiang Wei, Tingting Wu, Lei Wang, Jiani Cao, Ka Li, Hongling Zhao and ShuaiShuai Niu critically read and revised the manuscript.

## Funding

This work was supported by grants from the National Key R&D Program of China (Grant/Award Numbers: 2023YFC3605100 and 2023YFC2308600) and the National Natural Science Foundation of China (Grant/Award Numbers: 82472621 and 82171422).

## Data Availability

Data sharing is not applicable to this article as no datasets were generated or analysed during the current study.

## Appendix A

Cell viability assay: cell counting method

### Instruments and Equipment

(A.1.1) Microscope (magnification 4×, 10×, 20×, 40×).

(A.1.2) Blood count plate.

### Reagent

Unless otherwise specified, all reagents are of analytical grade, and the detection water is deionised water.

(A.2.1) Phosphate buffer: pH 7.4.

(A.2.2) 0.4% trypan blue dye solution.

### Test Steps

(A.3.1) Cell Suspension Preparation:

Collect the cells to be detected, digest the cell spheres into single cells or smaller cell spheres, or resuspend neurospheres (including 1–20 cells), prepare the cell suspension with phosphate buffer (A.2.1) and dilute to the appropriate concentration. The number of cells in each 1 mm^2^ square should be 20–50 cells. If it is higher than 200 cells, dilution is required.

(A.3.2) Cell Staining:

Mix trypan blue staining solution (A.2.2) and cell suspension (A.3.1) at a volume ratio of 1:1 and let stand for 30 s.

(A.3.3) Cell Count:

Cover the coverslip on the counting tank of the blood cell counting plate (A.1.2), take 10 μL of the mixed solution (A.3.2) and drop it on the edge of the coverslip in the counting chamber on one side, and take another 10μL of the mixed solution and drop it on the edge of the coverslip in the counting chamber on the other side, so that the mixed solution is filled between the coverslip and the counting plate, and stand for 30 s. Place the counting plate under the microscope (A.1.1) to count the stained cells and the total number of cells, respectively.

For the 16 × 25 counting chamber, take four 1 mm^2^ middle cells (i.e., 100 small cells) from the upper left, upper right, lower left and lower right according to the diagonal position. For the 25 × 16 counting chamber, count the 5 middle cells (i.e., 80 small cells) in the upper left, upper right, lower left, lower right and centre according to the diagonal position. When encountering cells on the large grid, generally only count the cells on the upper and left lines of the large grid (or only count the cells on the lower and right lines).

### Calculation and Analysis

(A.4.1) The cell survival rate is calculated according to the following formula:

$$S=\left(M-D\right)/M\times 100\%$$

In the formula, *S*—cell survival rate; *M*—total number of cells; *D*—number of stained cells.

Cell viability was the average of two samples. Calculate the average value of the results of cell survival rate counted twice and record it as the average cell survival rate.

### Precision

The absolute difference between the results of two independent determinations obtained under repeatability conditions shall not exceed 10% of the arithmetic mean.

## Appendix B

Cell marker protein detection immunofluorescence staining counting method.

### Instruments and Equipment

(B.1.1) Fluorescence microscope or confocal microscope. The excitation light waves are 488 nm and 594 nm.

(B.1.2) Horizontal centrifuge.

(B.1.3) Electronic balance.

### Reagent

(B.2.1) The experimental water is the first‐class water specified in gb/t 6682.

(B.2.2) Phosphate buffer: pH 7.4.

(B.2.3) Paraformaldehyde (PFA): purity 95%.

(B.2.4) Bovine serum albumin (BSA): purity ≥ 98%.

(B.2.5) Polyethylene glycol octyl phenyl ether (Triton X‐100).

(B.2.6) Anti‐human ptf1a and ascl1 antibodies, corresponding fluorescent secondary antibodies and antibodies that stain nuclei.

(B.2.7) Washing solution, fixation solution, blocking permeabilization solution, antibody diluent: prepare according to the corresponding requirements using electronic balance (B.1.3).

(B.2.8) Anti‐fluorescence quencher.

### Sample Storage

Human spinal cord GABAergic precursor cells and further cultured mature human spinal cord GABAergic interneurons were washed and fixed, respectively, and stored at 2°C–8°C. The fixative solution shall be put into the subpackaging container, sealed and marked, and shall be stored according to the relevant reagent instructions. The relevant antibodies were stored according to the instructions.

### Test Steps

(B.4.1) Sample Preparation and Fixation:

Neurospheres were collected and digested for 7–10 min to obtain a neurosphere suspension with an average of about 50 cells per neurosphere. The neurosphere suspension was seeded on Matrigel treated cell slides (round cell slides suitable for 24 well plates with a diameter of 14 mm) and cultured for 1–3 weeks. Discard the supernatant, add an appropriate amount of fixative solution and place it on ice for 20 min, and then wash with an appropriate amount of washing solution for 3–5 times, 3–5 min each time.

(B.4.2) Sealing and Permeability:

Incubate with blocking permeabilization solution on ice for 30 min, and then wash with washing solution.

(B.4.3) Antibody Incubation:

Dilute according to the antibody instructions.

(B.4.4) Washing:

Discard the blocking permeabilization solution, and wash the round cell slide three times with washing solution, each time for 5–10 min.

(B.4.5) Stained Nuclei:

Discard the washing solution, dilute it according to the instructions of antibody to stained cell nuclei, and then wash it according to (B.4.4).

(B.4.6) Sealing:

Add 10 μL anti‐fluorescence quenching blocking agent dropwise to each slide, clip the edge of the round cell slide and gently buckle it on the slide to avoid bubbles.

(B.4.7) Microscope Photography and Imaging:

The fluorescence microscope or confocal microscope was used to image the samples, and the exposure time of the fluorescence channel was adjusted according to the signal intensity of the negative samples for fluorescence imaging.

(B.4.8) Counting:

(B.4.8.1) Count the antibody positive cells that stain the nucleus in the photos of different batches and different fields, count the number of cells with antibody positive to the corresponding cell marker protein, and calculate the positive proportion of cell marker protein. The positive proportion is as follows:

P=b/h×100%

where *P*—positive ratio of cell marker protein; *H*—total number of antibody positive cells staining nuclei; *B*—total number of cells positive for antibody to cell marker protein.

### Result Analysis

The test results obtained are comprehensively analysed by the software. Refer to the software instructions for details.

## Appendix C

Whole cell patch clamp for cell function detection.

### Instruments and Equipment

Whole cell patch clamp apparatus and recording system.

### Reagent

(C.1.1) Artificial cerebrospinal fluid: 127 mM NaCl, 1.9 mM KCl, 1.4 mM MgSO_4_, 1.2 mM KH_2_PO_4_, 26 mM NaHCO_3_, 2.2 mM CaCl_2_‐2H_2_O, 10 mM glucose, pH 7.2, 282 mOsm.

(C.1.2) Electrode filling solution: 121 mM K ‐gluconate, 20 mM KCl, 10 mM HEPES, 4 mM M g‐ATP, 10 mM BAPTA, pH 7.4, 293 mOsm.

### Test Steps

(C.2.1) Sample Preparation:

Human spinal cord GABAergic neural precursor cells were seeded into the culture plate with climbing slices, and the mature human spinal cord GABAergic interneurons were formed after 3 weeks of continuous culture with neurotrophic factor rich medium.

(C.2.2) Sample Processing:

The climbing piece with cultured cells was transferred to the recording chamber filled with artificial cerebrospinal fluid for whole cell patch clamp recording.

(C.2.3) Test Method:

The electrode filling liquid fills the glass capillary pipette, close to the cell surface, and the instrument provides negative pressure to form a seal. Voltage clamp with a holding potential of −70 mV. Current clamp with a holding current of 0 pA. The resistance of the electrode was 10–20 mΩ. Spontaneous action potentials of cells were recorded in voltage clamp mode.

(C.2.4) Result Analysis:

The whole cell patch clamp instrument and clampift analysis software were used to record and analyse the waveform of spontaneous action potential of cells. If the cells showed a constant frequency of tonic discharge mode, they were human spinal cord GABAergic interneurons, and if the cells showed a non‐tonic discharge mode, they were other neuronal cells.

